# The Anabolic Androgenic Steroid Nandrolone Decanoate Disrupts Redox Homeostasis in Liver, Heart and Kidney of Male Wistar Rats

**DOI:** 10.1371/journal.pone.0102699

**Published:** 2014-09-16

**Authors:** Stephan P. Frankenfeld, Leonardo P. Oliveira, Victor H. Ortenzi, Igor CC. Rego-Monteiro, Elen A. Chaves, Andrea C. Ferreira, Alvaro C. Leitão, Denise P. Carvalho, Rodrigo S. Fortunato

**Affiliations:** 1 Laboratório de Radiobiologia Molecular, Instituto de Biofísica Carlos Chagas Filho, Universidade Federal do Rio de Janeiro, Rio de Janeiro, Brazil; 2 Laboratório de Biologia do Exercício, Escola de Educação Física e Desportos, Universidade Federal do Rio de Janeiro, Rio de Janeiro, Brazil; 3 Laboratório de Fisiologia Endócrina Doris Rosenthal, Instituto de Biofísica Carlos Chagas Filho, Universidade Federal do Rio de Janeiro, Rio de Janeiro, Brazil; 4 Polo de Xerém/Laboratório de Fisiologia Endócrina Doris Rosenthal, Instituto de Biofísica Carlos Chagas Filho, Universidade Federal do Rio de Janeiro, Rio de Janeiro, Brazil; Universidad Pablo de Olavide, Centro Andaluz de Biología del Desarrollo-CSIC, Spain

## Abstract

The abuse of anabolic androgenic steroids (AAS) may cause side effects in several tissues. Oxidative stress is linked to the pathophysiology of most of these alterations, being involved in fibrosis, cellular proliferation, tumorigenesis, amongst others. Thus, the aim of this study was to determine the impact of supraphysiological doses of nandrolone decanoate (DECA) on the redox balance of liver, heart and kidney. Wistar male rats were treated with intramuscular injections of vehicle or DECA (1 mg.100 g^−1^ body weight) once a week for 8 weeks. The activity and mRNA levels of NADPH Oxidase (NOX), and the activity of catalase, glutathione peroxidase (GPx) and total superoxide dismutase (SOD), as well as the reduced thiol and carbonyl residue proteins, were measured in liver, heart and kidney. DECA treatment increased NOX activity in heart and liver, but NOX2 mRNA levels were only increased in heart. Liver catalase and SOD activities were decreased in the DECA-treated group, but only catalase activity was decreased in the kidney. No differences were detected in GPx activity. Thiol residues were decreased in the liver and kidney of treated animals in comparison to the control group, while carbonyl residues were increased in the kidney after the treatment. Taken together, our results show that chronically administered DECA is able to disrupt the cellular redox balance, leading to an oxidative stress state.

## Introduction

Anabolic-androgenic steroids (AAS) are synthetic molecules similar to the male sex hormone testosterone. The classical therapeutic uses of these substances are the treatment of hypogonadism, bone marrow failure syndromes, bone mineralization and some muscle–wasting disorders [1]. The use of AAS to enhance physical performance or appearance has greatly increased, and individuals usually take doses 10 to 100 fold higher than the therapeutical dose; this abuse can cause many adverse effects [2].

Hepatic structure and function are severely altered by high AAS doses [1]–[3]. Serum levels of the hepatic enzymes aspartate-aminotransferase, alanine-aminotransferase and lactate-dehidrogenase are increased, and more severe disorders can be induced by high AAS administration, such as peliosis hepatis, hepatocellular hyperplasia and hepatocellular adenoma [1]–[3]. In the heart, AAS abuse increases the risk of cardiovascular diseases, possibly due to increased total cholesterol and low density protein levels, decreased high density lipoprotein levels, increased blood pressure, thrombosis, myocardial infarction and heart failure [4], [5]. The kidney is also affected: creatinine, blood urine nitrogen and uric acid are increased in AAS-abusers. Moreover, AAS users have a high risk of developing Wilm's tumor, which is otherwise not common in adults [6]–[8].

Liver, heart and kidney pathophysiology are usually linked to oxidative stress, which is characterized by a disruption of redox signaling and control. Reactive oxygen species (ROS), such as superoxide and hydrogen peroxide (H_2_O_2_), can be formed by xanthine-oxidase, cytocrome P-450 or mitochondrial electron transport chain, as a by-product, or directly by the NADPH oxidase (NOX) family of enzymes [9]. The NOX family is composed of seven members, NOX1-NOX5 and DUOX1/2, which are differentially expressed among tissues. The physiological roles of NOXs are quite diverse, they act in a wide range of cellular processes, such as cellular proliferation, calcium release and hormonal biosynthesis, but their overexpression is associated with the pathophysiology of various diseases [10]. Intracellular ROS levels are maintained at adequate levels by antioxidant systems that react with these molecules producing less reactive compounds. Catalase and GPx are involved in H_2_O_2_ detoxification, producing H_2_O directly or in a GSH-dependent reaction, while superoxide-dismutase catalyzes the conversion of superoxide to H_2_O_2_ [11].

As the majority of side effects elicited by AAS have their etiology linked to oxidative stress, the aim of this study was to evaluate the redox balance of AAS target tissues, such as liver, heart and kidney, in rats chronically treated with supraphysiological doses of nandrolone decanoate.

## Materials and Methods

### Experimental group

Adult male Wistar rats weighing 200–250 g were maintained in an animal room with controlled lighting (12-h light-dark cycle) and temperature (23–24°C). This investigation conforms to the Guide for the Care and Use of Laboratory Animals published by the US National Institutes of Health (NIH Publication No. 85–23, revised 1996) and was approved by the Institutional Committee for Evaluation of Animal Use in Research (Comissão de Ética com o Uso de Animais (CEUA) em Experimentação Científica do Centro de Ciências da Saúde da Universidade Federal do Rio de Janeiro, number: IBCCF 159). The animals were divided into two groups: normal control rats (submitted to vehicle injection; peanut oil with 10% of benzoic alcohol) and rats treated with nandrolone decanoate (Deca Durabolin (50 mg/mL (Organon)) 1 mg.100 g^-1^ b.w. Steroid and vehicle were administered by a single intramuscular injection in the hind limb once a week for 8 wk. We have previously reported that this treatment was effective, decreasing testicular weight and increasing serum testosterone levels in male Wistar rats [12], [13]. One week after the last injection, the rats were killed by decapitation, and trunk blood was immediately collected. After collection, the blood was centrifuged (15 min, 3000 g) and serum was collected and stored at −20°C. Liver, kidney and heart were excised, immediately frozen in liquid nitrogen and stored at −80°C. It is important to note that the tissues analyzed here are from the same rats utilized in Frankenfeld et al. (2014) study, in which we shown the effectiveness of the treatment (13).

### H_2_O_2_ generation measurement

H_2_O_2_ generation was quantified in heart, liver and kidney particulate fractions by the Amplex red/horseradish peroxidase assay (Molecular Probes, Invitrogen), which detects the accumulation of a fluorescent oxidized product. In order to obtain the microssomal fraction, the homogenates from liver, kidney or heart samples were centrifuged at 3000×g for 15 min at 4°C. Then, the supernantant was centrifuged at 100 000×g for 35 min at 4°C and the pellets were resuspended in 0.5 ml 50 mM sodium phosphate buffer, pH 7.2, containing 0.25 M sucrose, 2 mM MgCl_2_, 5 mg/ml aprotinin and 34,8 mg/ml phenylmethanesulfonyl fluoride (PMSF) and stored at −20°C until H_2_O_2_ generation measurements, as follows. The microssomal fraction was incubated in 150 mM sodium phosphate buffer (pH 7.4) containing SOD (100 U/ml; Sigma, USA), horseradish peroxidase (0.5 U/ml, Roche, Indianapolis, IN), Amplex red (50 µM; Molecular Probes, Eugene, OR), 1 mM EGTA in the presence or absence of 1 mM NADPH. The fluorescence was immediately measured in a microplate reader (Victor X4; PerkinElmer, Norwalk, CT) at 30°C, using excitation at 530 nm and emission at 595 nm [14]. Specific NADPH Oxidase activity was calculated by the differences between the activities in the presence and absence of NADPH.

The specific enzymatic activity was expressed as nanomoles H_2_O_2_ per hour per milligram of protein (nmol.h^−1^.mg^−1^). Protein concentration was determined by the Bradford assay [15].

### Real-time polymerase chain reaction analysis

Total RNA was extracted from the heart using the RNeasy Fibrous Tissue Mini Kit (Qiagen, Valencia, California), and RNeasy Plus Mini Kit (Qiagen, Valencia, California) was used for liver and kidney tissues, following the manufacturer's instructions. After DNAse treatment, reverse transcription was followed by real-time polymerase chain reaction (PCR), as previously described (16). GAPDH was used as an internal control. The specific oligonucleotides listed in Table 1, were purchased from Applied Biosystems (Foster City, California).

**Table 1:** Primers used for the Real-Time Polymerase Chain Reaction Assay.

	Forward	Reverse	Tm (°C)	Gene accession number (Ensembl)	Product size	Position of the primer
NOX1	AACTGGCTGTACTGCTTG	ATTCGTCCATCTCTTGTTCCAG	79.93	NM_053683	198	Forward: 15553–15570 (24383) Reverse: 17230–17251 (24383)
NOX2	CCATTCACACCATTGCACATC	CGAGTCACAGCCACATACAG	79.99	NM_023965	181	Forward: 536–556 (3556) Reverse: 757–776 (3556)
NOX4	TCCATCAAGCCAAGATTCTGAG	GGTTTCCAGTCATCCAGTAGAG	78.33	NM_053524	362	Forward: 165909–165928 (179656) Reverse: 178835–178854 (179656)
DUOX1	GATACCCAAAGCTGTACCTCG	GTCCTTGTCACCCAGATGAAG	81.76	NM_153739	196	Forward: 30616–30626 (34361) Reverse: 32106–32126 (34361)
DUOX2	TGCTCTCAACCCCAAAGT	TCTCAAACCAGTAGCGATCAC	84.35	NM_024141	191	Forward: 5181–5198 (18941) Reverse: 5891–5911 (18941)
GAPDH	TGATTCTACCCACGGCAAGT	AGCATCACCCCATTTGATGT	81.30	NM_017008	124	Forward: 2929–2948 (5076) Reverse: 3113–3132 (5076)

### Antioxidant enzymes activity

Heart, liver and kidney samples were homogenized in 5 mM Tris-HCl buffer (pH 7.4), containing 0.9% NaCl (w/v) and 1 mM EDTA, followed by centrifugation at 750×g for 10 minutes at 4°C. The supernatant aliquots were stored at −70°C. Catalase activity was assayed following the method of Aebi (1984) and was expressed as units per milligram of protein (U.mg^−1^) [17]. Glutathione peroxidase (GPx) activity was assayed by following NADPH oxidation at 340 nm in the presence of an excess of glutathione reductase, reduced glutathione and tert-butyl hydroperoxide as substrates [18], and expressed as nmol of oxidized NADPH per milligram of protein (nmol.mg^−1^). Total superoxide dismutase activity was determined by reduction of cytochrome C at 550 nm [19].

### Measurement of total protein reduced thiol and carbonyl residues

The oxidative damage of the studied tissues was determined by the measurement of total protein reduced thiols and carbonyl residues. Total reduced thiols were determined in a spectrophotometer (Hitachi U-3300) using 5,5-dithionitrobenzoic acid (DTNB). Thiol residues react with DTNB, cleaving the disulfide bond to give 2-nitro-5-thiobenzoate (NTB^−^), which ionizes to the NTB^2−^ di-anion in water at neutral and alkaline pH. The NTB^2−^ was quantified in a spectrophotometer by measuring the absorbance at 412 nm, and was expressed as nmol of reduced DTNB/mg protein [20]. Protein carbonyl residues was evaluated based on a reaction with dinitrophenylhidrazine (DNPH), as previously described by Levine et al. [21], by absorbance at 370 nm, using a molar absorption coefficient of 22,000 M^−1^cm^−1^, and was expressed as carbonyl derivates (carbonyl nmol/mg protein).

### Serum transaminases

Serum transaminases AST/OGT (Aspartate Aminotransferase/Oxalacetic Glutamic Transaminase) and ALT/TGP (Alanine Aminotransferase/Glutamic Pyruvic Transaminase) were measured by commercially available kits (Laborlab, No. 00300 and No. 00200, respectively).

### Statistical analyses

The results are expressed as the mean ± SEM. All data were analyzed by unpaired t test using the Graphpad Prism software (version 5, Graphpad Software, Inc., San Diego, USA). A value of p≤0.05 was considered statistically significant.

## Results

### NADPH Oxidase activity and mRNA levels

**Figure 1:**
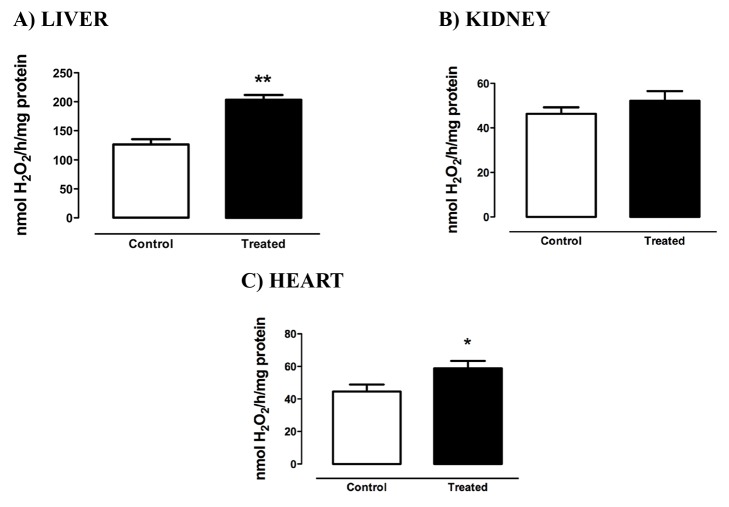
NADPH Oxidase activity in liver (A), kidney (B), and heart (C) of rats treated with Deca Durabolin (50 mg.mL^−1^ Organon, 1 mg/100 g bw, im), once a week, for 8 weeks. H_2_O_2_ production was determined in the microssomal fraction by the Amplex Red/Horseradish Peroxidase assay. Data were obtained with 10 animals from at least two independent experiments and are shown as mean ± SEM. * p<0.05; **p<0.001.

H_2_O_2_ generation was significantly greater in liver and heart tissues of the treated animals, as shown in figures 1A and 1C, respectively. No significant differences were found in kidney tissue (Fig. 1B).

**Figure 2:**
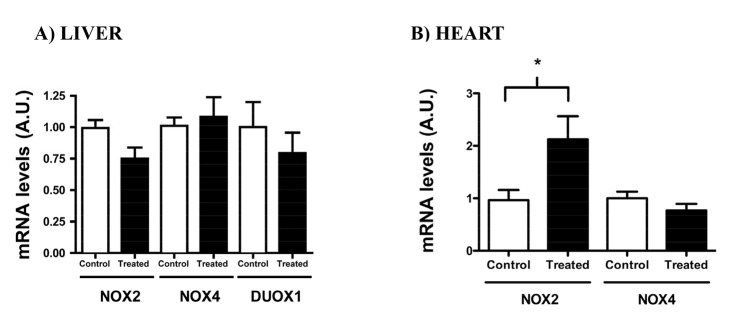
NADPH Oxidases mRNA levels in liver (A) and heart (B) of rats treated with Deca Durabolin (50 mg.mL^−1^ Organon, 1 mg/100 g bw, im), once a week, for 8 weeks. mRNA levels were determined by real-time PCR and was expressed relative to the control group. Data were obtained with 10 animals from at least two independent experiments and are shown as mean ± SEM. * p<0.05.

In order to evaluate the source of the higher H_2_O_2_ generation, we evaluated the mRNA expression of the NOX enzymes in the heart and the liver. While NOX 1, 2, 4 and DUOX 1 and 2 are found in the liver, NOX1, NOX2, NOX4 are expressed in the cardiovascular system. NOX2 mRNA levels were higher in the heart of the treated group in comparison to its control, but no differences were found in hepatic NOX2 and DUOX1 mRNA nor in the levels of the NOX4 mRNA in heart and liver (Fig. 2A–B). In our conditions, we could not detect the mRNA of NOX1 and DUOX2 in the liver and NOX1 in the heart.

### Antioxidant enzymes activity

**Figure 3:**
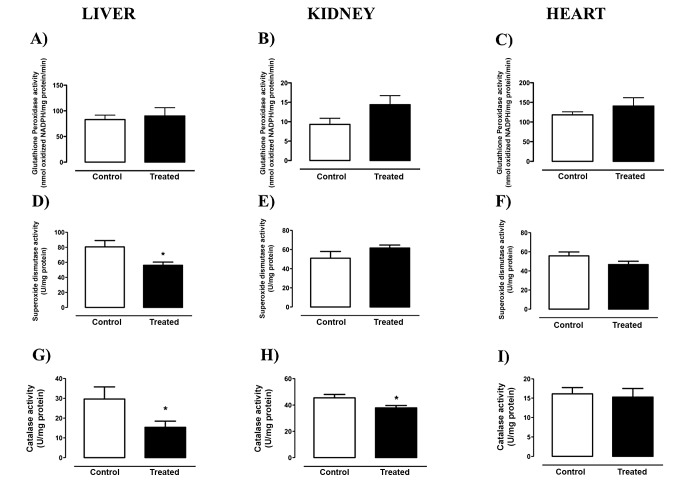
Antioxidant enzymes activities of rats treated with Deca Durabolin (50 mg.mL^−1^ Organon, 1 mg/100 g bw, im), once a week, for 8 weeks. Glutathione Peroxidase (A,B,C), Superoxide Dismutase (D,E,F) and Catalase (G,H,I) activities were measured in liver, kidney and heart homogenates, respectively, by spectrophotometry. Data were obtained with 6 animals from at least two independent experiments and are shown as mean ± SEM. * p<0.05.

Hepatic SOD (Fig. 3D) and Catalase (Fig. 3G) activities of the treated animals were decreased in comparison to the control group, but there was no change in GPx activity (Fig. 3A). In the kidney, the nandrolone treatment only decreased the catalase activity (Fig. 3H), and no differences in the cardiac antioxidant enzymes activities were found.

### Biomarkers of oxidative stress

**Figure 4:**
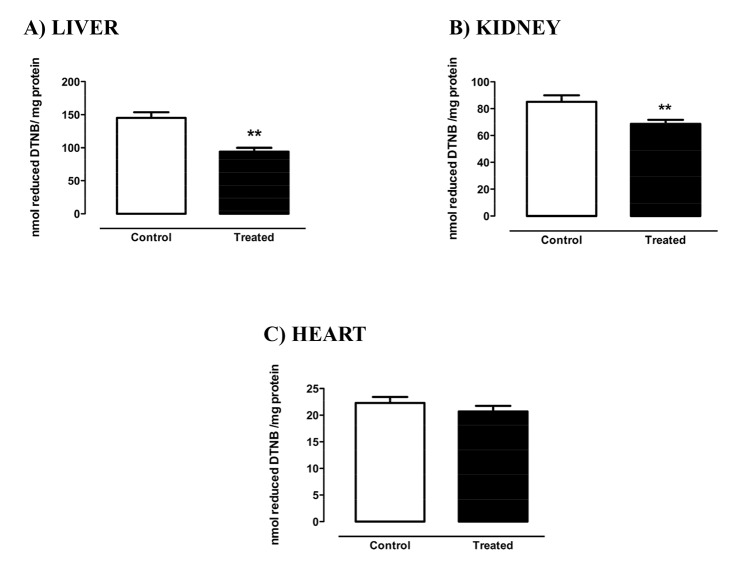
Reduced thiol content of liver (A), kidney (B), and heart (C) of rats treated with Deca Durabolin (50 mg.mL^−1^ Organon, 1 mg/100 g bw, im), once a week, for 8 weeks. Total sulfhydryl groups were measured by the reaction of thiols with DTNB, evaluated in a spectrophotometer at 412 nm. Data were obtained with 6 animals from at least two independent experiments and are shown as mean ± SEM. * p<0.05; **p<0.001.

**Figure 5:**
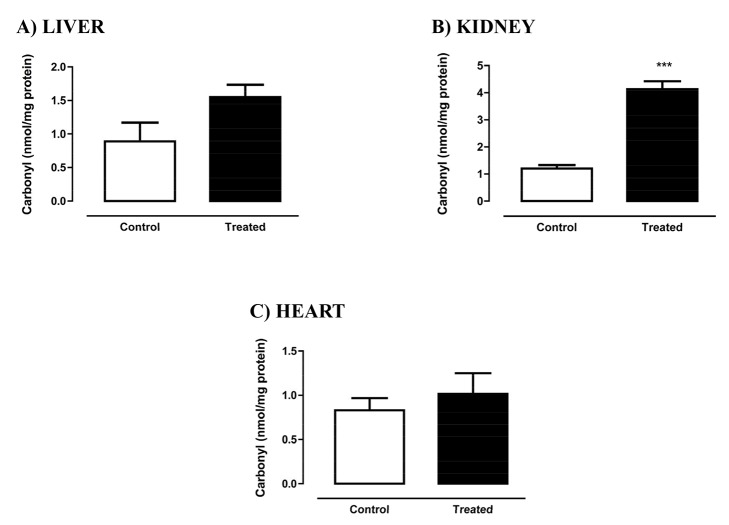
Protein carbonyl content of liver (A), kidney (B), and heart (C) of rats treated with Deca Durabolin (50 mg.mL^−1^ Organon, 1 mg/100 g bw, im), once a week, for 8 weeks. Protein carbonyl content was evaluated based on a reaction with dinitrophenylhidrazine (DNPH), evaluated in a spectrophotometer at 370 nm. Data were obtained with 6 animals from at least two independent experiments and are shown as mean ± SEM. * p<0.05; **p<0.001.

Thiol residues are mainly found in proteins and in low-molecular-mass metabolites such as the highly abundant glutathione (GSH), and can be reversibly oxidized by ROS to nitrosothiols or sulfenic acids, decreasing its cellular levels. On the other hand, carbonyl groups can be formed by the oxidation of proteins by ROS. As shown in figure 3, the protein thiol residues were significantly decreased in liver (Fig. 4A) and kidney (Fig. 4B) of the treated rats, but no changes were found in the heart (Fig. 4C). In the kidney, protein carbonyl content was significantly higher in the treated group as compared to its control (Fig. 5B), but no differences were found in the heart (Fig. 5C) or liver (Fig. 5A).

### Serum transaminases profile

No differences were seen between groups on the serum activity of AST/TGO (C = 58.41±3.77 U/L, n = 10; T = 58.93±3.51 U/L, n = 10) and ALT/TGP (C = 12.53±1.03 U/L, n = 10; T = 10.13±0.748 U/L, n = 10).

## Discussion

ROS are normally produced by virtually all cells of living organisms, and are able to act in the redox-dependent regulation of different cellular functions, including response to stressors, angiogenesis, cell proliferation and other [22]. In order to maintain intracellular ROS at adequate levels, antioxidant systems react with these molecules producing less reactive compounds. Conceptually, an imbalance between pro-oxidant compounds and antioxidant defenses leads to oxidative stress, but this concept has recently been redefined as the “disruption of redox signaling and control” [11]. ROS molecules can avidly react with cellular constituents. Thus, an increase in its generation or a decrease in its detoxification leads to increased ROS availability that can cause oxidative modifications of DNA, proteins, and lipids. These structural changes in biomolecules can alter cellular function and processes, and play an important role in a range of common diseases and degenerative conditions [11]. As the liver is the major organ involved in the drug metabolization, and the kidneys are responsible for its excretion, these organs are generally affected by high doses of AAS. On the other hand, the effects of AAS on the heart are well documented by us and others [23]–[25]. As a large body of evidence shows a role of oxidative stress in liver, kidney and heart dysfunction, we investigated if nandrolone decanoate, the most used AAS by bodybuilders and recreational athletes, could interfere in the redox balance of these tissues.

The most prominent changes caused by nandrolone decanoate treatment were seen in the liver. While NOX activity was increased, the antioxidant enzymes SOD and catalase were decreased after the nandrolone treatment. Moreover, total reduced thiol residues were decreased in nandrolone-treated rat liver. Pey et al. (2003) reported an increase in lipid peroxidation, as well as SOD, GPx and catalase activities in the liver of rats treated with stanozolol (a non-sterified) for 8 weeks, and concluded that in their model the overproduction of ROS exceeded the increase of antioxidant enzymes, leading to lipid peroxidation, but ROS generation was not evaluated [26]. Although the meaning of these results is in the same direction of our findings, there is a discrepancy in antioxidant enzymes activity found in our two studies, which could be due to differences in the AAS and dose utilized. The decreased levels of total hepatic reduced thiol residues in the treated rats suggest an increase in oxidative stress, since excess ROS can react with reduced thiols oxidizing them. Liver NOX2 and NOX4 mRNA levels were unaffected by the treatment, but the activity of these enzymes is not only affected by their mRNA/protein expression, but also by other factors such as protein association and activation by intracellular pathways [10]. So, our hypothesis is that NOX activity is increased, without changes in its expression. The kidney redox balance was also affected by AAS treatment, judging by the increase in protein carbonyl content and decrease of total reduced thiol residues, and diminished catalase activity. No significant changes were observed in the renal NOX activity, but we cannot exclude ROS overproduction by other enzymes, since we measured the activity of NOX enzymes. Cardiac antioxidant enzymes were not affected by treatment, but NOX2 mRNA levels and H_2_O_2_ production were higher after nandrolone decanoate administration. Utilizing the same treatment protocol, we have reported no differences in rat heart antioxidant enzymes, such as SOD, GPx and catalase, after nandrolone decanoate administration [27]. Interestingly, although we detected increased ROS production in the heart from DECA-treated animals, the reduced thiol residue level and protein carbonyl content were not changed. Thus, we may speculate that there was an increase in antioxidant defense mechanisms, other than modulation of SOD, GPx and/or catalase activities.

Our results clearly show that supraphysiological doses of nandrolone decanoate administered chronically are able to disrupt redox metabolism in the studied tissues, characterizing an oxidative stress state. A link between oxidative stress and DNA damage has been well demonstrated, but the subcellular localization where ROS are generated is crucial in this process [28]. NOX enzymes are transmembrane proteins and can be located at nuclear membrane, near to DNA, increasing the possibility of its damage [10]. Recently, Pozzi et al. (2012) evaluated the DNA damage elicited by nandrolone decanoate in liver, heart, kidney and peripheral blood in rats utilizing a single dose of 5 or 15 mg/Kg b.w. They used the alkaline comet assay, which detects DNA single and double strand breaks and alkali-labile lesions, and found an increased DNA damage in the liver and heart with the lower and higher dose, but only the higher dose increased DNA damage in the kidney, with values much lower than the other studied tissues [29]. Our results suggest that through the increase in ROS availability, nandrolone decanoate exposure can also lead to DNA damage, but more studies are necessary to elucidate this question. In fact, oxidative stress is implicated not only in carcinogenesis, but also in the pathogenesis of a wide range of diseases that can affect liver, heart, kidney and other tissues. AAS abuse is involved in liver tumor development and inflammation, cardiac autonomic dysfunction and hypertrophy, in addition to mesangial matrix accumulation and increased heat shock proteins in the kidney [23], [30], [31]. Interestingly, all dysfunctions cited above can be caused by an increase in ROS availability, and NOX enzymes seem to play an important role in those processes. In conclusion, we demonstrated that supraphysiological doses of nandrolone decanoate are able to disrupt redox balance in liver, heart and kidney. Many diseases are linked to oxidative stress, so we believe that this study helps to clarify the physiological changes caused by the abuse of this and related drugs, which increased notably in the past three decades. Besides, our results contribute to elucidate AAS side effects in order to warn AAS abusers about the problems associated with this practice.
